# Post-traumatic growth and influencing factors among parents of premature infants: a cross-sectional study

**DOI:** 10.1186/s40359-023-01360-7

**Published:** 2023-11-10

**Authors:** Fang Wang, Shuo Zhang, Chunyan Liu, Zhihong Ni

**Affiliations:** 1 Children’s Hospital of Soochow University, No. 92, Zhong nan St, Suzhou, 215025 Jiangsu China; 2 School of Nursing, Soochow University, Suzhou, Jiangsu China

**Keywords:** Cross-sectional studies, Neonatal intensive care unit, Premature infants’ parents, Post-traumatic growth

## Abstract

**Background:**

Post-traumatic growth is a positive psychological change that may aid recovery in individuals experiencing trauma. Owing to the lack of research in the area of parental care for premature infants, we decided to explore the levels and factors influencing post-traumatic growth among parents of premature infants in neonatal intensive care units. We believe that these findings will help reassess existing care practices so that healthcare providers can promptly identify negative emotions and take necessary measures to help develop the potential to enhance post-traumatic growth.

**Methods:**

A cross-sectional survey was conducted using convenience sampling between February and September 2022. Data were analysed using independent sample *t*-tests and one-way analysis of variance (ANOVA). Bivariate correlations were analysed using the Pearson’s or Spearman’s method, and related factors were analysed using multiple linear regression. We followed the SRQR checklist throughout the study period.

**Results:**

A total of 217 patients were effectively treated, with a recovery rate of 98.64%. Univariate analysis showed that the length of hospital stay, presence of only one child, parents’ age, marital status, education level, working status, and per capita monthly familial income were influencing factors. Bivariate analysis showed that post-traumatic growth was moderately and positively correlated with perceived social support, rumination, and family resilience. Multiple linear regression showed that purposeful contemplation, family resilience, education, family support, age, and marital status entered into the regression equation and together accounted for 47.4% of the total variation.

**Conclusions:**

It is necessary to pay attention to post-traumatic growth and familial stability in these families, provide aid in building a good support system, and encourage parents to mobilise their family and favourable factors to increase post-traumatic growth levels.

## Background

Premature birth is defined as the termination of pregnancy before 37 weeks of gestation [1]. According to the World Health Organization (WHO), approximately 15 million premature babies are born annually worldwide. The incidence of premature births ranges from 5 to 18% in 184 countries [2] and continues to increase [3]. Currently, the number of premature babies born annually in China is 1,172,300, accounting for 7.81% of global annual premature births. Additionally, China has the second highest premature birth rate worldwide. Owing to the continuous introduction of birth policies, the premature birth rate is expected to accelerate [2]. As the proportion of older mothers increases, the likelihood of pregnancy complications increases, contributing to an increase in premature birth rates. Premature birth is the primary cause of neonatal death. Premature birth-related complications cause one million deaths annually, accounting for more than 50% of neonatal deaths [3]; moreover, the mortality rate of premature infants is 12 times that of full-term infants [4]. In recent years, mortality rates have decreased due to the development of advanced medical technology, improvements in perinatal care programmes, and the establishment of neonatal intensive care units. However, premature birth results in a shortened gestation time, insufficient foetal nutritional reserve, dysplasia of various body systems, and weakened postnatal immune function. Compared to full-term infants, premature infants experience problems such as insufficient lung maturity, unstable blood flow, neurodevelopmental delay, and fragile blood vessels, leading to low resistance to the external environment after birth and a higher risk of complications [5]. Furthermore, short- and long-term sequelae of premature birth affect adult mortality [6]. Most premature babies require admission to Neonatal Intensive Care units (NICUs) because of their vulnerable physical condition; the specialised care in NICUs promotes organ maturation, enhances immunity, and improves long-term survival. In addition to inducing various complications, the frequent medical operations required for premature infants incur huge costs. Thanh et al.’s retrospective analysis found that although premature infants accounted for only 7% of all newborns, the total medical costs incurred for their care were as high as 37% [7]. Premature infants consume more health resources in terms of initial hospitalisation, rehospitalisation, outpatient services, and medical treatment in the first year after birth [7, 8]. Unstable physiological conditions, unpredictable disease development, and high medical costs are significant sources of stress that can lead to familial trauma [9]. In addition, stressors such as parental role change, parent-child separation, lack of knowledge of diseases, and high medical costs greatly impact parents’ psychology. During this period, their needs often remain unmet, leading to various negative emotions, such as anxiety, depression, perceived vulnerability, and post-traumatic stress disorder [10, 11].

Psychology directs and promotes behaviour. Previous studies have focused on examining negative psychological effects on individuals; however, in recent years, the impact of positive psychological experiences has also been considered. Parents of premature infants often experience pressure because they bear primary responsibility for this adverse and unexpected event. Moreover, caregivers’ reflection and growth after the event directly affect children’s coping ability and disease prognosis owing to their young age. Premature birth threatens the lives and health of mothers and children, cause familial trauma, and seriously affects parental post-traumatic growth (PTG). Negative emotions associated with trauma hinder PTG, and post-traumatic stress disorders may occur in severe cases. Post-traumatic stress disorder is common among parents of children in NICUs, and severe physiological conditions and uncertain prognosis of the foetus after birth aggravate its occurrence, hindering treatment and growth in children [12]. Parental PTG is crucial for facilitating early response in hospitals, the growth and development of premature infants after discharge, and family recovery. Parents of premature infants may experience helplessness and worry due to the closed medical environment and lagging disease knowledge during hospitalisation, as well as the potentially high risk of complications after discharge. Because parents are the main caregivers of newborns after discharge, continuous psychological problems can affect their mental health and seriously hinder self-perception of their roles and caring ability [13, 14]. This is not conducive to the recovery of the family and can affect the growth and development of premature infants, thereby forming a vicious circle.

To cope with the changes in circumstances before and after discharge, parents moved from focusing on the pain caused by the event to thinking about possible causes and ways to solve problems; subsequently, their values and priorities changed. They reestablished relationships with friends, family, and medical staff, thereby receiving external psychological, material, and social support to cope with traumatic events. Throughout the process, they demonstrated active medical cooperation and a strong demand for knowledge. Furthermore, a family’s ability to cope with external stressors plays an important role in positive parental psychology and behaviour, effectively reducing the family’s worries and improving the efficiency of action. Positive psychological and behavioural changes lead to parental PTG. In addition to the physical and mental health of family members, PTG levels are closely associated with the prognosis of premature infants. Low PTG levels affect the development of premature infants, and a poor prognosis causes familial trauma; these two factors influence each other. Currently, most patients studied are adults with cancer [15], and most studies on parental PTG have focused on cancer [16], autism [17], and congenital diseases [18]. Limited studies exist on children and their caregivers; consequently, investigations on PTG in parents of premature infants remain insufficient. Therefore, this study aimed to investigate the status quo and influencing factors of PTG in parents of premature infants treated in the NICU; explore the correlation between PTG levels and rumination, perceptive social support, and family resilience; promote the clinical development of targeted intervention measures; and make full use of favourable factors to improve parents’ mental state. Overall, we aimed to improve the prognosis of children and their families.

## Method

### Study design

A cross-sectional survey was conducted to explore the levels and factors influencing PTG among parents of premature infants in NICUs. This study followed the STROBE checklist for cross-sectional studies. This study was approved by the Medical Ethics Committee of Soochow University Children’s Hospital (2021C5196). The principles of informed consent and confidentiality were adhered to. Prior to the study, the parents of premature infants were informed of the purpose, significance, and precautions of the study, and informed consent was obtained. All questionnaires were anonymous, properly maintained, and used only for this study.

### Data collection and participants

This non-experimental investigative study was conducted at an advanced children’s hospital in Suzhou, China. The investigation lasted for eight months, beginning in February 2022. Based on the convenience sampling method, 217 subjects who met the sample scheduling standards were selected and signed informed consent forms were obtained. Premature infants meeting the following criteria were included: (1) gestational age < 37 weeks; (2) parents as the main caregivers; (3) diagnosis of three or more hospital diseases; (4) parents with normal cognitive and understanding abilities; and (5) parents who voluntarily participated in the questionnaire survey and study. We excluded samples from premature infants with congenital malformations or genetic disorders, and from families with major traumatic events within the first month of birth. This study adopted an offline investigation, and 20 samples were selected for pre-investigation in the early stages of testing its feasibility. The contents of the questionnaire were modified and improved, and the survey was conducted on the day the premature infants were discharged. After routine health education, questionnaires were distributed to parents, and informed consent was obtained. The investigator explained the significance and purpose of this study in detail. To ensure the integrity and reliability of the data, investigators checked each item, communicated it in a timely manner with parents, and supplemented missing items. A total of 220 questionnaires were sent out and 217 were effectively received, resulting in an effective participation rate of 98.64%.

### Instruments

Based on the purpose of this study, we developed an anonymous questionnaire to collect data through a review of relevant published literature. A pilot study involved pre-surveying 20 parents of preterm infants prior to a formal investigation to determine whether the questions were clear and understandable. The questionnaire included the following: (1) basic information of premature infants: sex, gestational age, birth weight, birth mode, length of hospital stay, etc.; (2) basic information of parents: sex, age, education level, religious belief, average monthly income, payment method, etc.; and (3) basic fertility information: whether the child was an only child, pregnancy, pregnancy complications, etc.

The Post-traumatic Growth Inventory (PTGI) is used to measure the experiences and growth of parents of premature infants after a traumatic event. In this study, the simplified Chinese version of the PTGI revised by Wang et al. (2011) was used, including 20 entries in five dimensions [19]. The index is scored on a six-point Likert scale. From “no such change was felt at all” to “a lot of such changes”, scores were respectively recorded as 0–5 points, with a total score of 0–105 points. Higher scores indicated higher PTG levels. A score of < 60 was considered low level, 60–65 as medium level, and ≥ 66 as high level. The reliability of the scale was good, with a total Cronbach’s α coefficient of 0.874; the Cronbach’s α coefficients of each dimension were 0.611–0.796.

The Chinese Event-Related Rumination Inventory (C-ERRI) was used to evaluate the cognitive processing styles and levels of parents of premature infants experiencing traumatic events. In 2011, Dr. Cann compiled a questionnaire based on PTG theory [20], and in 2013, Dong translated and revised the questionnaire, applying it to patients with accidental trauma [21]. The questionnaire consisted of 20 items in two dimensions (purposeful and intrusive meditation). Using a four-point Likert scale, the occurrence of such thoughts after trauma ranges from “never” to “often” on a scale of 0–3, with a total score of 0–60. The total score was proportional to the rumination level. The Cronbach’s α coefficient of the total scale was 0.92, that of intrusive meditation was 0.93, and that of purposeful meditation was 0.85; this was close to the reliability of the original scale.

The Perceived Social Support Scale (PSSS), developed by Zimet, was used to assess parents’ subjective perceptions and satisfaction with external social support [22]. The scale contains three dimensions and 12 items. Answers are scored on a seven-point Likert scale (1–7), from " slightly agree” to “strongly agree”, with the total score being 12–84 points. The score obtained is proportional to the level of social support. The Cronbach’s α coefficient of the scale was 0.922, while those for the two dimensions were 0.851 and 0.913, indicating good reliability.

The Family Resilience Assessment Scale (FRAS) evaluates families’ ability to use their own potential and tap surrounding resources in the face of negative events. It was developed by Dr. Sixbey, an American scholar, based on the family resilience model, and has been widely used in research in children with chronic diseases [23]. This scale was Sinicised by Dong [24], after which it contained 44 items in four dimensions: family communication and problem-solving, using social and economic resources, maintaining a positive attitude, and giving significance to adversity. A four-point Likert scale (1–4) was used, ranging from “strongly disagree” to “strongly agree”, with a total score of 44–176; this score correlated positively with the level of family resilience. The Cronbach’s α coefficient of the total scale was 0.96, while that of each dimension was 0.70–0.97, consistent with the original scale.

### Data analysis

We used Epidata3.1 to input data, and SPSS26.0 was used for data analysis after a double check. Statistical significance was set at *p* < 0.05. For sociodemographic data, count data were described statistically as frequencies and percentages. Mean ± standard deviation was used to statistically describe normally distributed measurement data, while median or interquartile spacing was used to statistically describe measurement data not conforming to the normal distribution. Two independent sample *t*-tests were used for bivariate variables with normal distribution and homogeneity of variance, and a single-factor ANOVA was used for multivariate variables. The Mann–Whitney *U* test was used for binary variables with non-normal distributions, and the Kruskal–Wallis *H* test was used for multiple classification variables. In the bivariate analysis, Pearson’s correlation analysis was used to determine the relationship between PTG and rumination, perceived social support, and family resilience for normally distributed data, whereas Spearman’s correlation analysis was used for other cases. A multiple linear regression method was adopted with PTG as the dependent variable and statistically significant indicators in univariate and bivariate correlation analyses as independent variables. Independent variable factors were gradually added to the equation model, and only statistically significant factors were retained to determine the factors influencing PTG and the best model.

## Results

### Descriptive analysis of participants

A total of 220 questionnaires were sent for this study, of which 217 were effectively received, with a recovery rate of 98.64%. Two questionnaires were deemed invalid, and one was withdrawn voluntarily. As shown in Table 1, the male-to-female ratio in the premature infants was 1.237:1. More than two-thirds of births were by caesarean section, with the proportion being significantly higher than that of natural births. The gestational age of 30% of the premature infants was < 32 weeks, whereas the minimum gestational age was 26 weeks. Most birth weights ranged from 1500 to 2499 g. Most hospital stays lasted 20–60 days. The number of only children (104) was slightly lower.

**Table 1:** Univariate analysis of the post-traumatic growth of parents of premature infants in NICU (n = 217)

Variables	Number, percentage (%)	PTGI score ($\stackrel{-}{\varvec{x}}\pm \varvec{s}$)	Statistical values (t/F)	*P* value
Age			8.294	0.000
<20 20~ 30~ ≥ 40	3(1.4) 78(35.9) 125(57.6) 11(5.1)	32.33 ± 11.24 64.71 ± 16.38 62.50 ± 16.89 43.09 ± 22.65		
Marital status			3.542	0.000
married unmarried	207(95.4) 10(4.6)	62.81 ± 17.30 42.90 ± 18.78		
Educational background			13.746	0.000
Primary school and below Junior high school Senior high school Bachelor’s degree and above	2(0.9) 54(24.9) 74(34.1) 87(40.1)	13.50 ± 2.12 54.06 ± 16.98 61.88 ± 18.27 67.89 ± 14.39		
Working state			17.323	0.000
On the job resigned unemployed	176(81.1) 22(10.1) 19(8.8)	64.72 ± 16.32 43.09 ± 16.84 57.47 ± 18.93		
Average monthly income			4.827	0.000
<3000 3000~ 5000~ >10,000	12(5.5) 57(26.3) 93(42.9) 55(25.3)	44.17 ± 13.11 59.49 ± 17.37 61.81 ± 19.06 68.40 ± 13.57		
Length of stay			3.033	0.030
<20 20~ 40~ ≥ 60	87(40.1) 71(32.7) 39(18.0) 20(9.2)	60.03 ± 20.16 60.39 ± 16.30 63.31 ± 16.01 72.55 ± 11.43		
Only child			2.098	0.037
Yes No	104(47.9) 113(52.1)	64.52 ± 17.49 59.48 ± 17.86		

PTGI- Post-Traumatic Growth Inventory

### Univariate analyses of factors associated with PTG

We collected information regarding characteristics of the premature infants, families, and pregnancies of the research subjects. In the data analysis, we found statistically significant differences in some factors among the three aspects. The results of the data analysis showed statistically significant differences in PTG scores of premature infants in the NICU at different times of admission and whether they were only children (*p* < 0.05). Statistically significant differences were observed in PTG scores among parents of premature NICU infants of different ages, marital status, education level, working status, and per capita monthly family income (*p* < 0.05) (Table 1). In premature infants, there were no significant differences in sex, gestational age, birth weight, or Apgar scores. From a family perspective, there were no statistically significant differences between different family roles, residences, family structures, and religious beliefs. In terms of pregnancies and deliveries, the pregnancy and delivery models, adverse birth events, and pregnancy complications did not show statistically significant effects.

### PTG scores and correlation variables

In this study, the total PTG score of parents of NICU premature infants ranged from 12 to 100, with an overall average score of 61.89 ± 17.89 and dimensional mean score of 3.09 ± 0.89. There were 77 cases (35.5%) with a score < 60, 41 cases (18.9%) with a score of 60–65, and 99 cases (45.6%) with a score ≥ 66. From high to low, they scored life perception 19.50 ± 6.07, new possibilities 12.89 ± 3.80, self-transformation 10.33 ± 4.54, personal strength 10.23 ± 2.96, and relationship with others 8.89 ± 3.11. The total score of rumination was 20.08 ± 11.63, while the average score of items was 1.00 ± 0.58. The total score of understanding social support was 62.24 ± 12.15 points, while the average score of items was 5.19 ± 1.01 points. The total score of family resilience was 134.27 ± 15.62, while the average score of items was 3.05 ± 0.36 (Table 2).

**Table 2:** Total scores and subdimension scores of the PTGI、ERRI、PSSS and FRAS (n = 217)

Variables	Number of items	Dimension (mean ± SD)	Item (mean ± SD)
PTGI			
Total score Relating to others New possibilities Appreciation of life Personal strength Spiritual change	20 3 4 6 3 4	61.89 ± 17.89 8.89 ± 3.11 12.89 ± 3.80 19.50 ± 6.07 10.23 ± 2.96 10.33 ± 4.54	3.09 ± 0.89 2.96 ± 1.04 3.22 ± 0.95 3.25 ± 1.01 3.41 ± 0.99 2.58 ± 1.14
ERRI			
Total score Intrusive rumination Deliberate rumination	20 10 10	20.08 ± 11.63 9.60 ± 7.04 10.47 ± 6.21	1.00 ± 0.58 0.96 ± 0.70 1.05 ± 0.62
PSSS			
Total score Family support Friend support Other support	12 4 4 4	62.24 ± 12.15 22.06 ± 4.58 20.10 ± 4.63 20.08 ± 4.10	5.19 ± 1.01 5.52 ± 1.15 5.03 ± 1.16 5.02 ± 1.03
FRAS			
Total score Communicate and solve problems Use social resources Maintain a positive attitude Give meaning to adversity	44 27 8 6 3	134.27 ± 15.62 84.74 ± 10.10 23.00 ± 3.73 18.08 ± 2.71 8.45 ± 1.27	3.05 ± 0.36 3.14 ± 0.37 2.88 ± 0.47 3.01 ± 0.45 2.82 ± 0.42

PTGI – Post-Traumatic Growth Inventory; ERRI – Event-Related Rumination Inventory; PSSS – Perceived Social Support Scale; FRAS – Family Resilience Assessment Scale

### Bivariate analysis of PTG

Bivariate analysis revealed that PTG was moderately and positively correlated with perceived social support (*r* = 0.417, *p* < 0.01) as well as with rumination (*r* = 0.426, *p* < 0.01) and family resilience (*r* = 0.437, *p* < 0.01).

### Multiple linear regression analysis

The results of multiple linear regression showed that six variables were included in the regression equation: purposeful meditation, family resilience, education level, family support, age, and marital status (*R*^*2*^ = 0.489), with an adjusted *R*^*2*^ of 0.474 (Table 3). These variables accounted for 47.4% of the variation in PTG levels among the parents of premature NICU infants. The effects of each dimension on PTG were as follows: purposeful rumination (0.419); family resilience (0.260); educational level (0.136); family support (0.166); age (0.137); and marital status (0.106). The regression equation was as follows: PTG of parents of NICU premature infants = 6.328 + 1.203 purposeful meditation + 0.296 family resilience + 2.956 education level – 4.116 age – 8.966 marital status. A regression equation model was used to obtain *F* = 12.923 and *p* < 0.001.

**Table 3:** The influencing factors of PTGI by multivariable linear regression analysis

Variables	*β*	SE	*β’*	*t*	*P*
constant	6.328	10.662		0.593	0.553
Purposeful meditation	1.203	0.145	0.419	8.303	0.000
Family resilience	0.296	0.065	0.260	4.575	0.000
Degree of education	2.956	1.215	0.136	2.432	0.016
Family support	0.647	0.227	0.166	2.851	0.005
Age	-4.116	1.514	-0.137	-2.719	0.007
Marital status	-8.966	4.413	-0.106	-2.032	0.043

## Discussion

PTG is an important positive psychological indicator for parents of premature infants admitted to NICUs. In this study, the parents of premature infants were investigated and related factors were explored. The relationships between social support, rumination, family resilience, and PTG were also examined.

In our study, the total PTG score of parents of premature NICU infants was 61.89 ± 17.89, which was considered moderate. Both parents scored 3.09 ± 0.89 points in PTG entries, higher than the score of parents of children with accidental injuries (58.23 ± 14.23) [25], and lower than that obtained from parents of children with malignant bone tumours (64.92 ± 8.61) [26]. This may be because most premature births are diagnosed in advance and undergo medical monitoring, allowing parents time to prepare for the event. Furthermore, parents must actively learn and master the care of premature infants before discharge. Therefore, the PTG tended to reach its highest level and remained stable at discharge. Meanwhile, the prolonged course of malignant tumours and the high instability of the disease may lead to long-term high PTG levels in parents of children with bone cancer. In the current study, 77 (35.5%) parents had low PTG levels, 41 (18.9%) had medium PTG levels, and 99 (45.6%) had high PTG levels, suggesting that parents of premature infants in the NICU were generally stable; however, there were still deficiencies. The overall PTG scores of 111 fathers and 106 mothers were 62.05 ± 18.52, showing no significant difference between groups. Furthermore, the order of scores for each dimension, ranked from high to low, was life perception, new possibilities, self-transformation, personal strength, and relationships with others. This indicates that parents of premature NICU infants display weaknesses in personal strength and relationships with others during PTG. Possible reasons for this include relatively weak contact between hospitals and families during the pandemic, reduced communication frequency between parents and medical staff, and fewer opportunities for parents to understand and acquire knowledge and skills related to the disease.

We found that PTG levels were significantly higher in parents with higher levels of education. This may be because parents with low education levels lack adequate perception and understanding of the disease, producing disease uncertainty and fragile perception [27]. Parents with higher education levels can exert better initiative to learn about and understand disease progression [28]. A better understanding of the prognosis of disease development is required to respond to events with a positive attitude and manner, thus obtaining relatively high chances of growth. PTG levels were higher in parents aged 20–39 years and lower in parents aged < 20 and > 40 years. This is because young parents have less experience and are often overwhelmed, whereas older parents may not adapt easily because of slower thinking. Parents aged 20–39 years had richer life experiences, more stable social relations, strong social abilities, and good tolerance and adaptability to stressful events. In this study, parents aged 30 years and older of premature infants accounted for the largest proportion of participants (62.7%). This may be attributed to the adjustment of national fertility policies and social tendency towards late childbirth. After the two-child policy was relaxed, an increasing number of people chose to have children, and late marriages and births became more common in large cities. We observed higher PTG levels in married parents; further, a good marriage promotes harmony in family relations, and familial support contributes to an improvement in PTG levels [29]. Compared with married families, parents of premature infants in unmarried families face negative emotions caused by the lack of emotional support between spouses and the need to deal with the heavy financial burden and high pressure of care. This detrimentally affects the PTG levels.

We found that family support had a positive effect on PTG levels in parents of premature NICU infants. Material and spiritual support from family members is crucial for familial stability, and parents facing difficulties require emotional and informational support. Family support strengthens parents, enhances their sense of security and belonging, and stimulates positive psychological changes [30]. After experiencing traumatic events, parents of children attach more importance to the family’s role, cherish every member of the family, and realise the importance of mutual help and reliance among members [31, 32]. PTG is closely related to the family environment, and closeness and emotional expression among family members are the main influencing factors [33]. Purposeful rumination positively affected the PTG of parents of premature NICU infants. Proactive thinking is the process of finding and solving problems after a stressful event that can guide individuals to adapt to stress and improve recovery [34]. During the child’s hospitalisation, parents experienced separation anxiety in the early stages, and their state of isolation made them eager to participate in childcare. To cope with the circumstances during hospitalisation and after discharge, the parents of the child reflected on valuable events and emotional experiences in the past and considered their influence on the present and future. In addition, parental PTG levels increased with increased levels of family resilience. From the perspective of sharing internal resources as well as the acquisition and utilisation of external resources, family resilience is an important indicator of a family’s ability to recover from a traumatic event [35]. It evaluates families’ use of their potential and ability to exploit surrounding resources when facing negative events [36]. In addition, it helps family members quickly adapt to the occurrence and development of a disease and effectively cope with patient care, thus improving the prognosis and quality of life [18]. The more resilient the family, the better the parents are at finding and relying on resources in the family and the surrounding environment to aid in coping with the disease, which improves PTG levels.

### Limitations

Since a convenience sampling method was used to collect samples in this study, and the sample size was only collected in a children’s hospital, it may have led to a certain degree of selectivity bias, and the representativeness of the sample size needs to be improved. Post-traumatic growth is a dynamic process; therefore, the level and influencing factors of PTG at different nodes may differ. Next, we will expand the sample source and size, conduct a dynamic assessment, and explore multidimensional interventions to improve PTG in parents of premature infants.

## Conclusions

Parents of premature infants in the NICU experienced moderate to high levels of PTG, as seen in 64.52% of the study participants. Among the individual dimensions of growth, the single-item average score for life perception was the highest, and the single-item average score for relationships with others was the lowest. Factors influencing these scores included time of stay in the NICU, single-child status, parental age, marital status, education level, working status, family per capita monthly income, perceived social support, rumination, and family resilience. Purposeful meditation, family resilience, educational level, family support, age, and marital status were entered into the regression equation, accounting for 47.4% of the total variation. Among these factors, marital status had the greatest influence on PTG. Therefore, medical staff should pay more attention to the characteristics of PTG and familial stability of parents of premature NICU infants. Furthermore, they should focus on developing targeted nursing measures, improving parents’ psychological states, and promoting the prognosis of children and family health.

## Data Availability

The datasets analysed in the current study are available from the corresponding author on reasonable request.

## List of Abbreviations

ANOVA: One-way analysis of variance
ERRI: Event-Related Rumination Inventory
FRAS: Family Resilience Assessment Scale
FRAS: Family Resilience Assessment Scale
NICU: Neonatal intensive care unit
PSSS: Perceived Social Support Scale
PSSS: Perceived Social Support Scale
PTG: Post-traumatic growth
PTGI: Post-traumatic growth inventory
SRQR checklist: Standards for Reporting Qualitative Research
STROBE checklist: Strengthening the Reporting of Observational Studies in Epidemiology
WHO: World Health Organization

## Declarations

### Statistical considerations

The authors (s) affirm that the methods used in the data analyses are suitably applied to their data within their study design and context, and the statistical findings have been implemented and interpreted correctly.
